# Multifocal infections of the musculoskeletal system: description of a safe one-step procedure for eradication of associated spinal infections

**DOI:** 10.1186/1754-9493-7-30

**Published:** 2013-09-25

**Authors:** Anna Voelker, Nicolas H von der Hoeh, Jens Gulow, Sven Kevin Tschoeke, Christoph-Eckhard Heyde

**Affiliations:** 1 Department of Orthopaedic Surgery, University Hospital Leipzig, Liebigstrasse 20, 04103 Leipzig, Germany; 2 Department of Orthopaedic Surgery, Park Hospital Leipzig, Leipzig, Germany

**Keywords:** Multifocal infections, One-stage procedure, Spinal infection

## Abstract

**Background:**

The aim of this study was to evaluate the clinical outcome after radical surgical treatment of multifocal infections involving the spine.

**Methods:**

The study demonstrates a retrospective chart review of seven patients who had more than three different abscesses in the musculoskeletal system and at least one of them in the area of the spinal column. All patients had a sepsis.

**Results:**

Beside different musculoskeletal abscesses four patients had a spondylodiscitis in the cervical spine segments C4/5 or C5/6. Six patients had inflammatory processes in the lumbar spine with epidural abscesses, diffuse thoracolumbar paravertebral abscesses and a spondylodiscitis in different segments. In all cases we performed a radical surgical treatment of all related inflammatory focuses. Prompt radical surgical treatment of the spine included decompression, debridement and in the cases of spondylodiscitis a fusion of the involved segments. For more than one focus at the spine, a surgical one-step procedure was performed. An antibiotic therapy was administered for six to eight weeks. In follow up examinations no signs of ongoing inflammatory processes were seen in imaging studies or laboratory tests.

**Conclusions:**

In the event of multiple abscesses of the musculoskeletal system involving the spine an early correct diagnosis and radical surgical treatment is recommended. We strongly favor a surgical single-stage procedure for treatment of multiple infections of the spine. In addition to a radical debridement and a sufficient decompression, the segmental fusion of affected areas in spondylodiscitis is essential. At the same time a surgical therapy of all other infected sites should be performed.

## Background

Treatment of sepsis resulting from multifocal infections involving the spinal column is a challenge. Usually, patients with multiple inflammatory focuses have an immunodeficiency. This can be caused by a tumor disease or a metabolic disease [1,2]. Patients who are affected by rheumatism also have a higher risk of developing multiple abscesses. The underlying reasons are the immunosuppression of the specific medication or the disease itself [3]. In particular patients with total joint replacement and a predisposing illness requiring an immunosuppressive therapy have a higher risk for septic arthritis.

Pyogenic spondylodiscitis or spinal abscesses usually are caused by hematogenous spreading of pathogens from other infectious sites. Staphylococcus aureus followed by Streptococcus species and gram-negative bacteria are common pathogens [4].

Infections of the spine vary from paraspinal and epidural abscesses to discitis or spondylodiscitis. Secondary abscess formations in the paravertebral muscles or in the psoas muscles have been described [5]. Detecting secondary abscesses in the deeper muscle layers is a challenge because of unspecific inconstant local clinical symptoms. These usually range from no symptoms at all over local pain to neurological symptoms.

Without thorough imaging studies it is difficult to primarily detect all infectious sites. Depending on availability CT-scan [6], MRI [7] and PET-CT-scan [8] are recommended.

A variety of different surgical techniques for florid infections of the spine have been reported: minimal invasive procedures with debridement and decompression, posterior instrumentation with or without fusion, or combined dorso-ventral approaches in a single- or two stage procedure [9-11].

Next to a radical debridement of all infected tissue a subsequent specific therapy with antibiotics respective to the microbiological resistogram is necessary [12].

In our retrospective study, we present seven cases of multifocal infections of the musculoskeletal system involving the spine with sepsis.

The aim of our study was to evaluate the outcome of these patients after a radical surgical treatment of all foci was performed.

## Methods

The study demonstrates a retrospective chart review of seven patients with multiple focal infections involving the spine who were treated in our Departement of Orthopeadic Surgery from 03/2009 to 01/2012.

The collected personal data were age, gender, predisposing factors and associated illnesses.

Patients with more than three different abscesses in the musculoskeletal system and at least one of them near the spinal column were included in the study. All patients had a sepsis and underwent surgical treatment of the multifocal infections. The surgical procedure was determined individually for each patient depending on the localization of the infected site, number of abscesses and involvement of the surrounding tissue. Laboratory values including C-reactive protein (CRP) and white blood cell counts (WBC) were monitored during the hospital stay.

All patients were treated with antibiotics. The initial administration of broad spectrum antibiotics was adjusted if the microbiological resistogram of the intraoperatively taken probes made this necessary. The duration of initial i.v.-administration depended on the decrease of the inflammation values. Afterwards the adminstration was continued orally for a minimum total of 6 weeks and terminated only after verification of normal inflammatory values.

We analyzed the medical records, the imaging studies and follow up reports. Additionally we evaluated microbiological results from the intraoperatively taken probes.

Preoperatively the imaging studies including plain radiographs, CT-scans and MRI’s were evaluated to locate all infectious sites. In two cases a PET-CT scan was performed.

Clinical follow up 6 months up to 2 years after surgery included physical examinations, periodical tests of the inflammatory laboratory values (CRP and WBC), and radiological imaging.

Postoperatively plain radiographs of the spine were used for assessment of correct implant position, consolidation of fusion and the segmental alignment. MRI-studies of the spine were performed after 3–6 months for postoperative reevaluation of the previously inflamed processes.

## Results

The study included two male and five female patients with sepsis and multifocal infections involving the spine. The mean age was 65 years (54–78 years). The average hospital stay was 55,6 days (24–86 hospital days).

In five cases the primary site of infection could be detected. Three patients had an initial infection of a total knee prosthesis, one patient had an infection of a central venous port system and one patient had a purulent bacterial arthritis of the hip.

The predisposing factors and co-morbidities of the patients and the different medical conditions including the detected primary source of infection are shown in Table 1. Two patients had no specific underlying medical condition. Two patients suffered from rheumatoid arthritis which was being treated with immunosuppressives, cortisone and with a TNF alpha blocker (Etanercept or Adalimumab). The specific cortisone therapy was not halted during the hospital stay. To prevent a Mb. Addison crisis higher doses were administered intra- and postoperatively (day of operation: 100 mg, first day after surgery: 50 mg, second day after surgery: 25 mg cortisone intravenously). The therapy with Etanercept and Adalimumab was interrupted.

**Table 1:** Medical conditions in patients with multifocal infections including the spine

**Detected primary source of multifocal infections**	**Number of patients**
Infection after total knee replacement	3
Infection of a central venous port system	1
Bacterial arthritis of the hip	1
Unknown primary source of infection	2
**Predisposing factors and co**-**morbidities**	**Number of patients**
Rheumatoid arthritis with immunosuppressive therapy	2
Ductal carcinoma of the breast with a history of chemotherapy	1
Insulin dependent diabetes	2
Chronic renal failure	2

The medical history of two patients revealed an insulin-dependend diabetes and a chronic renal failure in two others. In one patient the underlying disease was a ductal carcinoma of the breast with a history of chemotherapy.

Different abscesses in the musculoskeletal system were found by radiological imaging with CT-scan, PET-CT scan and MRI’s. They varied from four to ten different sites. In addition to inflammatory processes of the spine we found septic arthritis of the glenohumeral joint, the elbow joint, the sternoclavicular joint, the knee joint, abscesses in the iliopsoas muscles, in the paravertebral muscles and in the gluteal muscles (Table 2).

**Table 2:** Locations of different inflammatory processes and abscesses

**Patients**	**Location of musculoskeletal inflammatory processes**	**Total number of inflammatory abscesses**
1	Intraspinal abscess L3-S3, lumbar paravertebral abscess, abscesses psoas musle left side, abscess sternoclavicular joint, gluteal muscles left side and area of greater ischiadic foramen	6
2	Spondylodiscitis C5/6 with epidural abscess, thoracolumbar paravertebral abscesses, abscess axillary region left side, infection of a central venous port system with subcutaneous abscess	4
3	Spondylodiscitis L3/4 with epidural abscess, bacterial arthritis of the hip, abscesses in the psoas muscles	4
4	Bacterial arthritis of metacarpophalangeal joints digitus II und III right, bacterial arthritis upper ankle joint both sides, infection of a total knee prosthesis both sides, bacterial arthritis glenohumeral joint both sides, abscess iliac muscle left side, spondylodiscitis C4 to C6	10
5	Infection of a total knee prosthesis left side, bacterial arthritis knee joint right, spondylodiscitis L1/2, abscess sternoclavicular joint right side	4
6	Spondylodiscitis C4/5 and L2 to L5, lumbar paravertebral abscess, abscesses in the psoas muscles both sides	5
7	Spondylodiscitis C5 to C7, lumbar paravertebral abscess and spondylodiscitis Th12/ L1, bacterial arthritis glenohumeral joint right, bacterial arthritis knee joint left, bacterial arthritis of the hip right, infection of soft tissue dorsum of the hand right	7

In all cases we performed a radical surgical treatment of all involved infectious sites. Depending on the localization of the infection, different procedures were necessary.

The joint infections were either treated by arthroscopic lavage or open debridement. In cases of septic arthritis after total knee replacement either a radical open synovectomy or removal of the implants in severe cases were performed.

All affected segments of the spine were treated operatively, preferably in a single-stage procedure. One patient preoperatively presented with weakness of the muscles innervated by the C5 nerve root and weakness of both legs resulting from a spondylodiscitis of the segment C4/5 and L2/3 with epidural abscesses.

Four patients had a spondylodiscitis in the cervical spine: One of them had a spondylodiscitis in the segment C4/5, one in the segments C5 to C7 with an epidural abscess and a prevertebral abscess, one in the segment C5/6 with an epidural abscess and one patient had an inflammatory process involving the cervical spine from C4 to C6.

**Figure 1:**
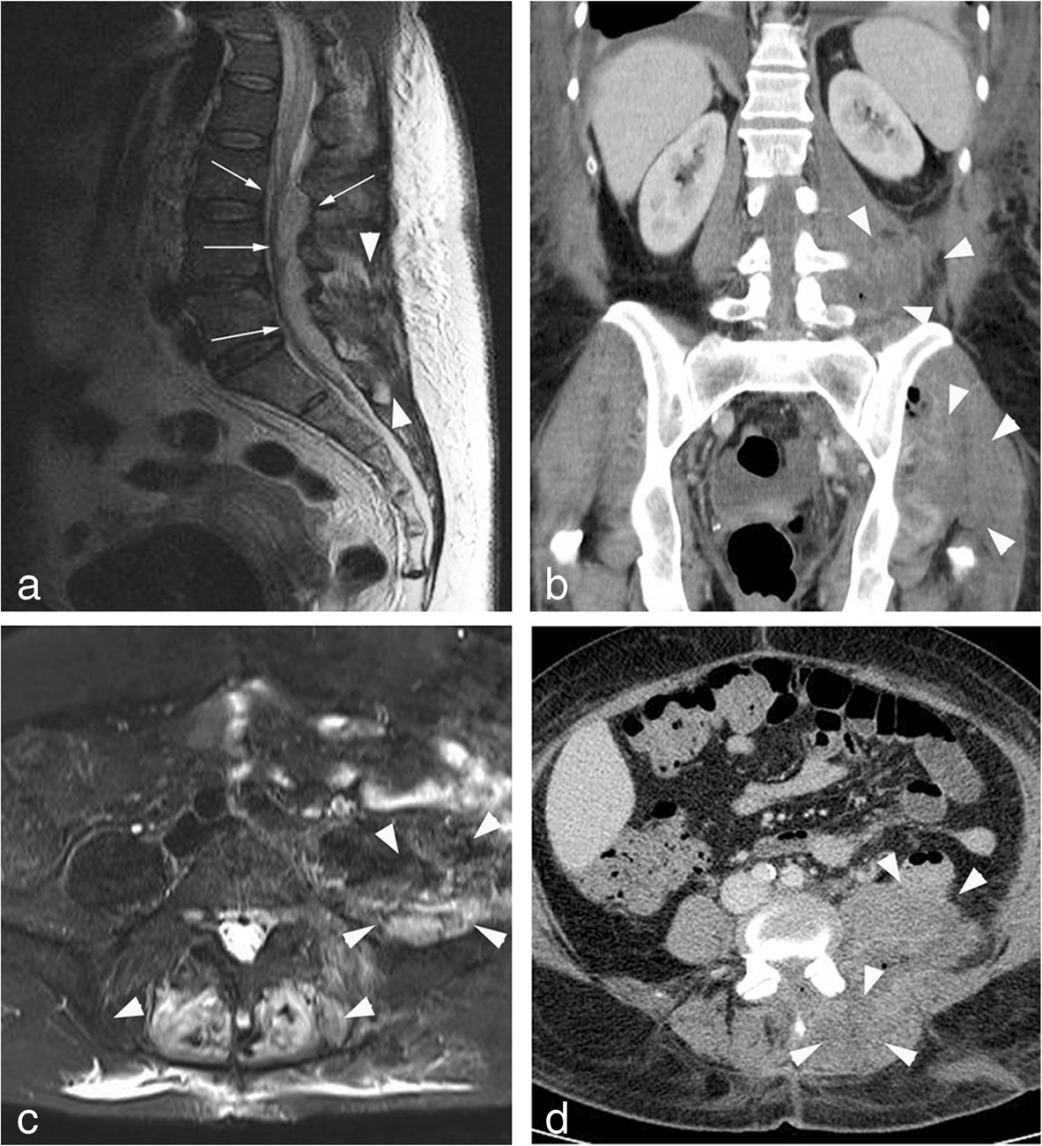
**Shows different inflammatory musculoskeletal sites in one patient: lumbar MRI with an epidural abscess (slim white arrows, a) as well as abscesses of the lumbar paravertebral muscles (bold white arrows, a).** CT-scan of the abdomen and pelvis and MRI of the spine demonstrating the psoas abscesses on the left side, inflammatory focus in the paravertebral muscles and abscesses in the gluteal muscles on the left side (bold arrows, **b**, **c** and **d**).

Six patients had a single or a second inflammatory processes in the lumbar spine: One epidural abscess ranging from L3 to S3, diffuse thoracolumbar paravertebral abscesses together with an epidural abscess in the lumbar spine, one spondylodiscitis L3/4 with an epidural abscess, one spondylodiscitis L1/2, one spondylodiscitis Th12/L1 with paravertebral abscesses in the lumbal muscles and one spondylodiscits involving the segments from L2/3 to L4/5 with an epidural abscess. Three of the patients had additional abscess formations in the psoas muscles (Table 3) (Figure 1).

**Table 3:** **Sites of spinal infections**, **stages of surgical treatment of the spine and bacteriology of tissue samples from the spine**

**Patient number**	**Site of spinal infection**	**Surgical treatment**	**Operative stages**	**Bacteriology of tissue samples**
1	Intraspinal abscess L3-S3, lumbar para-vertebral abscesses, abscesses psoas musle left side	Decompression and abscess evacuation L4-S1, open abscess drainage paravertebral lumbar, gluteal muscle and psoas musle left side	1	Staph. aureus
2	Spondylodiscitis C5/6 with epidural abscess, thoracolumbar abscesses paravertebral with epidural abscess	Tissue debridement Th10-S1 paravertebral, intraspinal abscess evacuation and decompression L3/4, debridement and fusion C5/6	2	negative
3	Spondylodiscitis L3/4 with epidural abscess, abscesses both psoas muscles	Posterior instrumentation L2-L5, decompression and debridement L3/4 and fusion, abscess evacuation psoas muscle	1	negative
4	Spondylodiscitis C4-C6	Corporectomy C5, bone grafting, plate fixation C4-C6	1	Staph. aureus
5	Spondylodiscitis L1/2	Posterior stabilization Th12-L3, decompression L1/2, debridement disc space L1/2 and fusion	1	E. coli
6	Spondylodiscitis C4/5 with epidural abscess, spondylodiscitis L2-L5 with epidural abscess, abscesses psoas muscles	Decompression, abscess evacuation, debridement disc space C4/5 and ventral spondylodesis C4/5, posterior stabilization Th12-L5, decompression L1-L4 and disc space debridement with fusion, abscess evacuation	1 (and CT-guided drainage abscesses psoas muscles)	Staph. aureus
7	Spondylodiscitis C5 to C7 with prevertebral and epidural abscess, spondylodiscitis Th12/L1, absesses in lumbar muscles	Decompression, abscess evacuation, debridement disc space C5 to C7 and ventral spondylodesis C5 to C7 and posterior lumbar stabilization Th11 to L2 with fusion Th12/ L1, tissue debridement Th12-L5 paravertebral	1	negative

Surgical treatment with decompression, debridement and in the cases of spondylodiscitis a fusion of the involved segments were performed. Tissue probes were taken from all infectious sites for microbiological testing.

Abscesses in the psoas muscles were debrided and drained either during the same surgical intervention or in one case percutaneously one day after surgery. All patients stayed at least one day in the intensive care unit.

In three cases we could detect Staphylococcus aureus and in one case Escherichia coli in the tissue samples from the spine (Table 3). In three cases the probes of the spine were negative for pathogens. In tissue samples from other sites of inflammatory tissue, different pathogens could be identified by microbiological studies, including: Klebsiella pneumonia, Vancomycin resistant enterococcus subspecies, Candida cruseii, Enterobacter amnigeus, Enterococcus faecalis and Streptococcus mites. Two patients additionally suffered a urinary tract infection induced by extended spectrum beta lactamase producing Enterobacteriaceae and Vancomycin resistant enterococcus subspecies.

The C-reactive protein and WBC were monitored for the entire hospital stay. In all cases the inflammatory laboratory parameters decreased after the radical surgical treatment.

**Figure 2:**
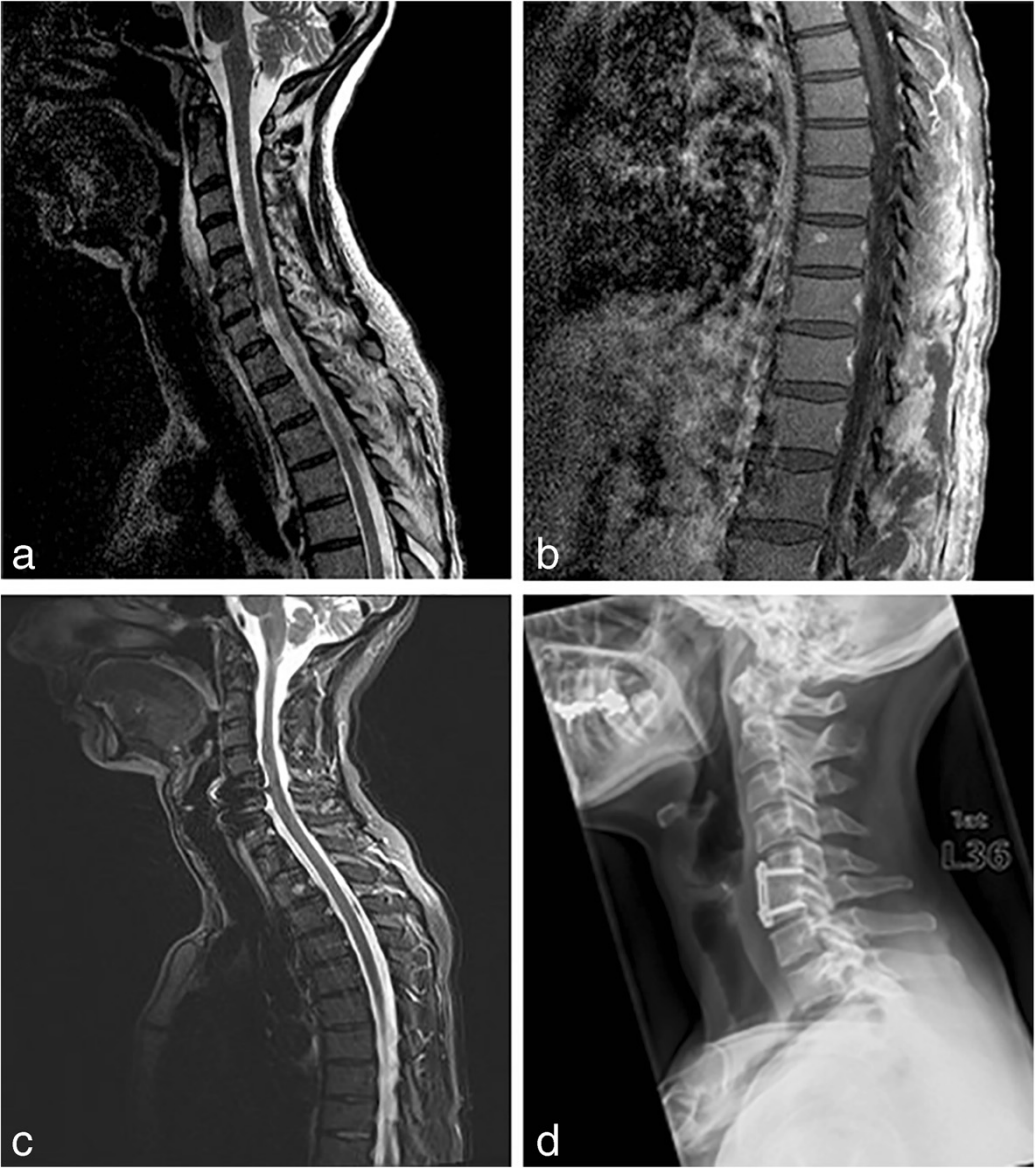
**MRI of one patient showing a spondylodiscitis C5/6 with an epidural abscess (a) and thoracolumbal abscesses of paravertebral muscles (b).** Follow up MRI with no signs of an ongoing inflammatory process **(c)** and control x-ray showing the consolidated fusion after ventral spondylodesis **(d)**.

**Figure 3:**
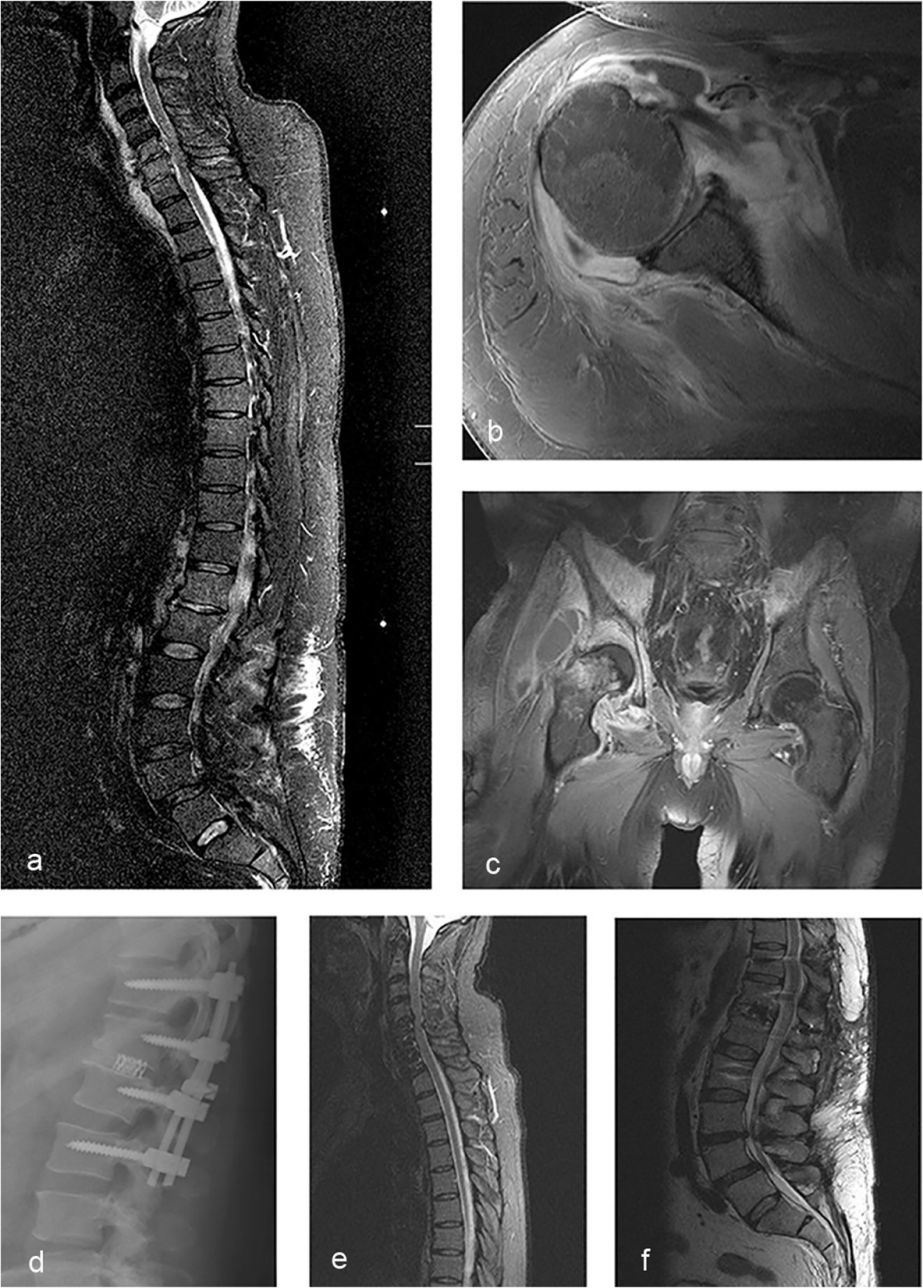
**Shows different pictures of one patient: MRI of total spine with spondylodiscitis C5-C7 with a prevertebral and epidural abscess and a spondylodiscitis Th12/L1 (a), bacterial arthritis of the right glenohumeral joint (b), bacterial arthritis of the right hip (c), control x-ray after posterior stabilization Th11-L2 with interbody fusion Th12/L1 (d), follow up MRI after ventral cervical spondylodesis and posterior lumbar stabilization with interbody fusion (e, f).**

All patients recovered well after radical surgical treatment. The patient with preoperative neurological symptoms showed no significant improvement of the neurological function after surgical intervention. After discharge all patients were seen every 14 days for at least eight weeks in our outpatient clinic for examination and control of the inflammatory laboratory values. Three to six month after surgical intervention one follow up MRI of the affected spine segments was conducted. All follow up imaging studies were evaluated by an independent radiologist. The MRI’s of the spine showed normal findings without ongoing inflammatory processes (Figures 2 and 3). All patients had normal inflammatory laboratory parameters at the end of outpatient treatment.

## Discussion

Sepsis resulting from multifocal infections of the musculoskeletal system is a life-threatening condition. Delayed treatment leads to a high morbiditiy and mortality. A timely indication for surgical treatment of all florid infectious sites is necessary especially in patients with beginning multiple organ failure [13].

A wide range of predisposing illnesses is commonly associated with multifocal infections of the musculoskeletal system e.g. rheumatoid arthritis, diabetes, tumors or immunosupressive therapy. Five of seven patients in our study had a predisposing illness (Table 1). Patients treated with biologica due to rheumatoid arthritis have a higher risk of septic arthritis. Galloway et al. investigated the influence of anti-tumor necrosis factor (TNF) therapy on septic arthritis and concluded that the risk for those patients to develop septic arthritis is doubled under this therapy [14].

Thorough imaging diagnostics including x-ray’s, CT-scan and MRI’s of the clinically infected areas have to be conducted. If possible a PET CT-scan is preferable for the evaluation of multiple florid infections. It is also useful in the case of spinal infections in which MRI has difficulties in differentiating infectious from degenerative end-plate abnormalities or postoperative changes [15].

Due to the PET-CT’s long duration of investigation it only plays a limited role for critically ill and septic patients who require immediate operative treatment as well as intensive care medicine.

The CT-scan is a highly sensitive and specific method to detect fluid accumulations in muscles e.g. psoas abscesses [16].

The MRI is the standard method in detecting florid infections of the spine. Imaging of the entire spine is absolutely recommended (Figure 3). Next to detecting inflammatory processes this method allows to rule out degenerative entities and tumors. Using different MRI-sequences helps to detect soft tissue involvement, involvement of the vertebral body and also helps to plan the extent of the surgical procedure [17].

Unspecific laboratory values like WBC and CRP were elevated in all patients preoperatively. However they do not correlate with the severity of an infection. In critically ill patients Procalcitonin [18] as well Interleukin-6 [19] are good parameters to monitor the effect of surgical and antibiotic therapy. However they are more expensive.

Isolating the pathogens is necessary for a specific antibiotic therapy. Blood cultures and intraoperatively taken probes are required. We recommend collecting swaps as well as tissue samples to reliably detect all different bacteria. A microbiological incubation should be performed at least for seven days. The antibiotic therapy should not be started before attaining tissue probes so that no false negative microbiological results are generated. Furthermore a specific antibiotic therapy can prevent the selection of multiresistant germs. In three patients in our study the detected pathogen in tissue probes from the spine was Staphylococcus aureus (Table 3). These findings are consistent with the literature where Staphylococcus aureus is the most frequently detected pathogen of spondylodiscitis next to Streptococcus species and gram-negative bacteria [4]. The fact that we could not isolate a specific pathogen in around 45 percent of the cases of spondylodiscitis or epidural abscess is a well known problem. The data in the literature differs greatly (10% to 40%) for negative microbiological tests of intra-operatively taken probes [20].

In these cases, calculated antibiotic therapy with a wide spectrum against gram positive and gram negative pathogens is required.

In our clinic intravenous antibiotic therapy is administered by peripheral intravenous cannulation or by central venous catheter (CVC). Peripherally inserted central catheters (PICC) are not necessary for treatment of multifocal infection of the musculoskeletal system. Most of the antibiotics can later be given orally except vancomycin and ceftriaxone. CVCs or PICCs have the risk of catheter-related blood stream infections, deep vein thrombosis and venous embolism [21,22]. We do not use PICC at all. Chopra et al. could show in a meta-analysis that PICCs are associated with a higher risk for deep vein thrombosis than CVCs [23]. The duration of i.v.-administration depended on the steady decrease of the inflammation values. Oral administration followed for a total of at least 6 weeks and was only terminated when the inflammatory values had normalized.

We recommend weekly controls of the inflammatory values and bi-weekly clinical examinations in the outpatient clinic.

Close cooperation of the surgeon with a microbiologist and an infectiologists helps to develop a specific treatment plan [24]. In specific questions an oncologists or rheumatologists can also be involved in the post-operative treatment.

Surgical techniques in treatment of spondylodiscitis or epidural abscesses include decompression of neurological structures, abscess evacuation, stabilization and re-alignment of the spine [20].

Neurological symptoms occur more often in cervical than in lumbar spondylodiscitis. One patient in our study presented with neurological symptoms of the innervated C5 dermatome and muscles along a weakness of both legs. She had a spondylodiscitis in the segment C4/5 with spinal narrowing and spondylodiscitis L1/2 with an epidural abscess. For treatment a sinlge-stage procedure with cervical ventral disc resection, abscess evacuation and fusion of the involved segment and decompression L1/2, L2/3 and L3/4, posterior stabilization Th12-L5 and interbody fusion L1 to L4 were performed. Previously reported studies strongly recommend early surgical treatment for cervical spondylodiscitis with decompression, debridement and stabilization because of the rapid increase of neurological symptoms in this sensitive area [25]. In the literature single-stage and dual-stage surgical procedures for surgical treatment in spondylodiscitis are described. We always prefer a single-stage procedure for spine surgery independent of how many areas of the spine are affected (Table 3). In one case we performed a two stage procedure. In the first operation the abscesses in the thoracolumbar paravertebral muscles were drained in an urgent operation of a septic patient. One day later the patient underwent a surgical debridement and fusion of the segments C5/6 in spondylodiscitis by a skilled spine surgeon, who was not available at the time of the first operation.

Puncture of a clinically diagnosed infected joint is the fastest and easiest way to confirm the diagnosis and obtain first microbiological probes for incubation [1]. The therapy of choice for joint infections, especially in patients with total surface implants is the radical debridement or removal of the implants in severe cases [26].

In spine surgery different implants like plates, titanium cages and rod systems are widely used in treatment of spondylodiscitis. Several studies demonstrate that these implants do not extend infections or induce a relapsing infection [9]. Even expandable titanium cages have no higher risk for persistent infections. Liljenqvist et al. investigated twenty cases of severe spondylodiscitis of the thoracal and lumbar spine that were treated with posterior stabilization and anterior fusion with expandable titanium cages. No persistent or recurrent spinal infection was reported in this study [27]. The previously reported data is consistent with our results. No reoperation of the spine had to be performed because of an ongoing infection.

Secondary psoas abscesses usually occur due to a spreading of infections located in the spinal colum, the gastrointestinal tract or urogenital tract [5]. In our study three patients developed psoas abscesses associated with lumbar spondylodiscitis or lumbar epidural abscesses. In two cases the psoas abscesses were drained intra-operatively with an open extraperitoneal approach and in one case drained CT-guided postoperatively. Abscess drainage is absolutely necessary together with surgical treatment of the primary source of infection. Drainage alone can lead to a recurrence of the local purulent infection [28].

## Conclusion

**Figure 4:**
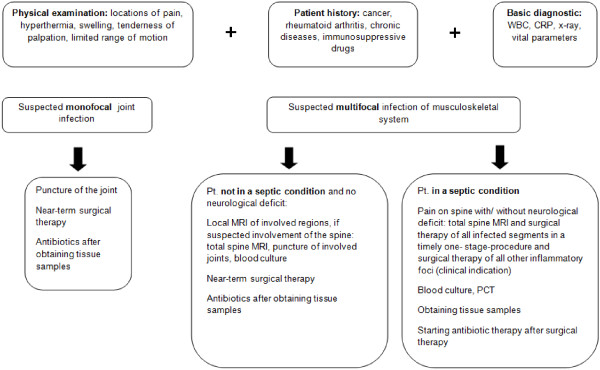
**Flow chart: evaluation and treatment of multifocal infections of the musculoskeletal system.**

Infections of the musculoskeletal system require a specific treatment. Next to a timely diagnosis of all infected sites the early indication for radical surgical treatment of all these sites is necessary (Figure 4). In the case of infected segments of the spine a radical debridement, a sufficient decompression and the segmental fusion in spondylodiscitis is essential. Although a radical single-stage surgical treatment in the area of the spine is a complex procedure and strenuous for the patient our results show that this kind of treatment has a good outcome.

At the same time surgical therapy of all other local abscesses and inflammatory focuses independent of their localization has to be performed. A specific antibiotic therapy needs to be administered and close follow up examinations need to be conducted.

### Consent

Patients were informed verbal and in written form and confirmed their approval on a consent form.

## Competing interests

All authors declared that they have no competing interests.

## Authors’ contributions

AV made substantial contributions for conception, design, analysis and interpretation of the data. NvdH did the main part of data acquisition. JG has been involved in drafting the manuscript. SKS has been involved in design and analysis of the data. CEH has given final approval of the version to be published. All authors read and approved the final manuscript.
